# Our Contemporaries

**Published:** 1912-07

**Authors:** 


### OUR CONTEMPORARIES

The undersigned publisher edited in this form a Monthly Bibliographical Review international journal for the whole literature of medicine with the collaboration of numerous savants from home and abroad, edited by the bbliographe H. Albert-Helimers. chief editor, annually 12 numbers (1 volume of 100—120 sheets); monthly 1 number of 8—10 printed sheets. Mk. 30.—.

The enorm value as far as concerns especially the second part (periodic literature) particularly will without a doubt being appreciated by every medical man. Actually it is not possible to the savants of medicine being informed exactly about all publications worth knowing, for the literature of medicine comprehends such an extended domain that a surgeon cannot know all that having been edited or being still published, even not in the special domain, which coming in question for the savants individually.

Also, he d'ont find in the special libraries of medicine all the principal periodicals necessaries being informed of all the new publications.

The "Monthly Bibliographical Review" edited by the undersigned publishing office gives the index of all the most important medical newspapers of the world.

The "Monthly Bibliographical Review" supplies a want considering that numerous societies of medicine have answered to the inquiry of the editor "that they would gladly patronise such a journal," "that the edition of such a mensual review would be a loudable enterprise," etc., etc.

The underwriter may with right pretend that a journal of the kind as edited by him does not exist in the whole world. Even the library of the Surgeon General's Office U. S. A. Army in Washington (the most important library of the world edited well an "Index Catalogue," but this gives only the new publications in books, phamplets, dissertation, periodicals, etc., but d'ont indicate the articles of the different journals of medicine.

The undersigned publisher would be very agreeable also, to get your order on the "Monthly Bibliographical Review" as soon as possible and remain, dear sir, with compliments, yours faithfully,

*Publisher of Monthly Bibliographical Review.*

RETTIG & KOLLMORGEN,

